# Supplementary material for: FERN – a Java framework for stochastic simulation and evaluation of reaction networks
Source: BMC Bioinformatics. 2008 Aug 29;9:356. doi: 10.1186/1471-2105-9-356 (PMC2553347; doi:10.1186/1471-2105-9-356)
Supplement: Additional file 1 — FERN distribution, Version 1.3. This archive contains the FERN source code and binaries as well as documentation and example models in FernML and SBML. [file 1471-2105-9-356-S1.zip › fern/doc/javadoc/fern/tools/functions/package-summary.html]

fern.tools.functions


---


|  |  |  |
| --- | --- | --- |
| **Overview** \| **Package** \| Class \| **Use** \| **Tree** \| **Deprecated** \| **Index** \| **Help** | |  |
| **PREV PACKAGE**   **NEXT PACKAGE** | **FRAMES**    **NO FRAMES**     **All Classes** |  |


---

## Package fern.tools.functions

Provides common function delegates and implementations.

**See:**
  
          **Description**

| **Interface Summary** | |
| --- | --- |
| **Probability** | Function delegate for conditional probabilities. |

| **Class Summary** | |
| --- | --- |
| **Probability.Constant** | Implementation that yields a constant (and hence independent) probability. |
| **Probability.ReactionProbability** | Implementation for the reaction probability for the autocatalytic network creation. |

## Package fern.tools.functions Description

Provides common function delegates and implementations.

---


|  |  |  |
| --- | --- | --- |
| **Overview** \| **Package** \| Class \| **Use** \| **Tree** \| **Deprecated** \| **Index** \| **Help** | |  |
| **PREV PACKAGE**   **NEXT PACKAGE** | **FRAMES**    **NO FRAMES**     **All Classes** |  |


---
